# CT and MR of recurrent primary cutaneous adenoid cystic carcinoma with multiple metastases

**DOI:** 10.1259/bjrcr.20150243

**Published:** 2016-11-02

**Authors:** Asik Ali Mohamed Ali, Praveen Sharma, Rujuta Rege, C Seena, N Kulasekaran, Saveetha Rajesh

**Affiliations:** ^1^Radiology and Imaging Sciences, Saveetha Medical College Hospital, Chennai, India; ^2^Cardiothoracic Imaging, Vancouver General Hospital, Vancouver, Canada

## Abstract

Primary cutaneous adenoid cystic carcinoma is a rare slow-growing neoplasm, with limited literature reporting the involvement of the scalp. It has a tendency to recur locally; however, lymph node, distant pulmonary and bony metastases are exceptionally rare. We highlight the case of a 65-year-old female with primary cutaneous adenoid cystic carcinoma with distant pulmonary and bony metastases and the importance of imaging in diagnosing distant metastasis and perineural spread.

## Clinical presentation/investigations

In July 2012, a 65-year-old female presented with a subcutaneous nodule in the left postauricular region. She had no history of previous skin lesions or systemic tumour disease. Excision biopsy performed elsewhere showed benign skin adnexal tumour–eccrine cylindroma. She did not undergo any further treatment. In October 2014, the size of the lesion increased; non-contrast CT performed elsewhere demonstrated a mass lesion in the left temporal region posterior to the pinna with erosion of the underlying mastoid bone cortex. Fine needle aspiration cytology of the lesion showed epithelial neoplasm. Excision of the lesion with mastoid exploration was performed. Histopathological examination of the operated lesion showed features of malignant neoplasm with perineural invasion, and a diagnosis of adenoid cystic carcinoma (ACC) was made. Subsequently, the patient underwent external beam radiotherapy with a dose of 5000 cGy for a period of 3 months. In April 2015, the patient came to the dermatology department in our hospital, with complaints of tenderness and swelling in the left preauricular region; she was hard of hearing on the left side. Physical examination showed that the swelling was non-mobile and firm in consistency. Audiological evaluation revealed left conductive hearing loss. CT and MRI examination was requested to evaluate recurrence.

## Imaging findings

**Figure 1.**
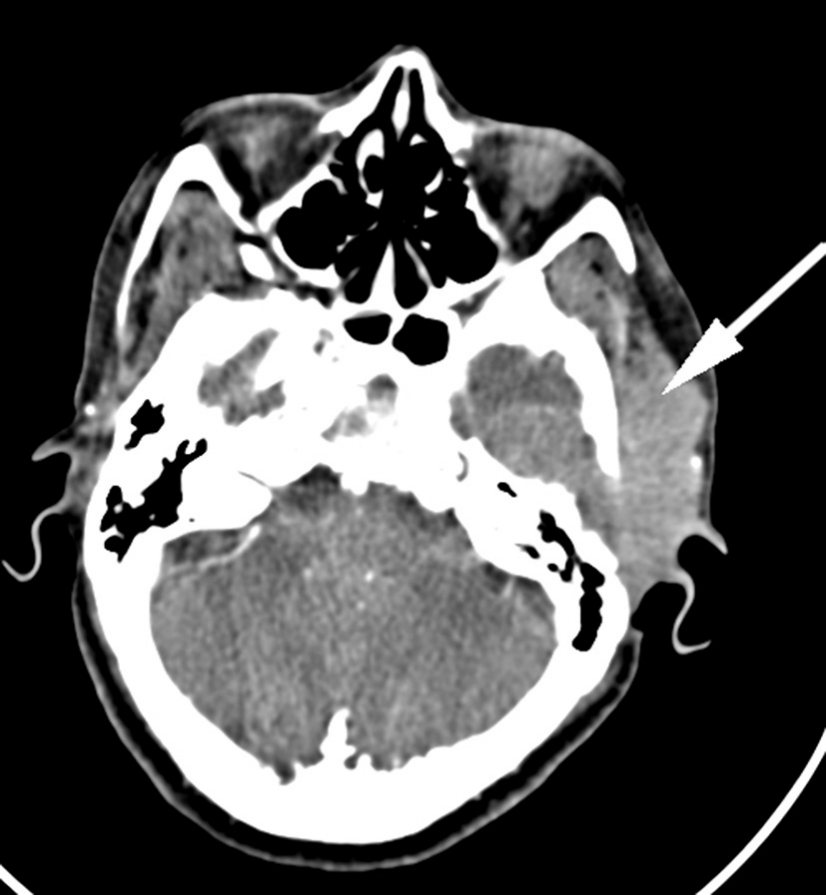
CT imaging of the neck shows an ill-defined homogeneously enhancing lesion in the left preauricular region of the scalp extending into the left masticator space causing erosion of the squamous part of the temporal bone with intracranial extension (arrow).

**Figure 2.**
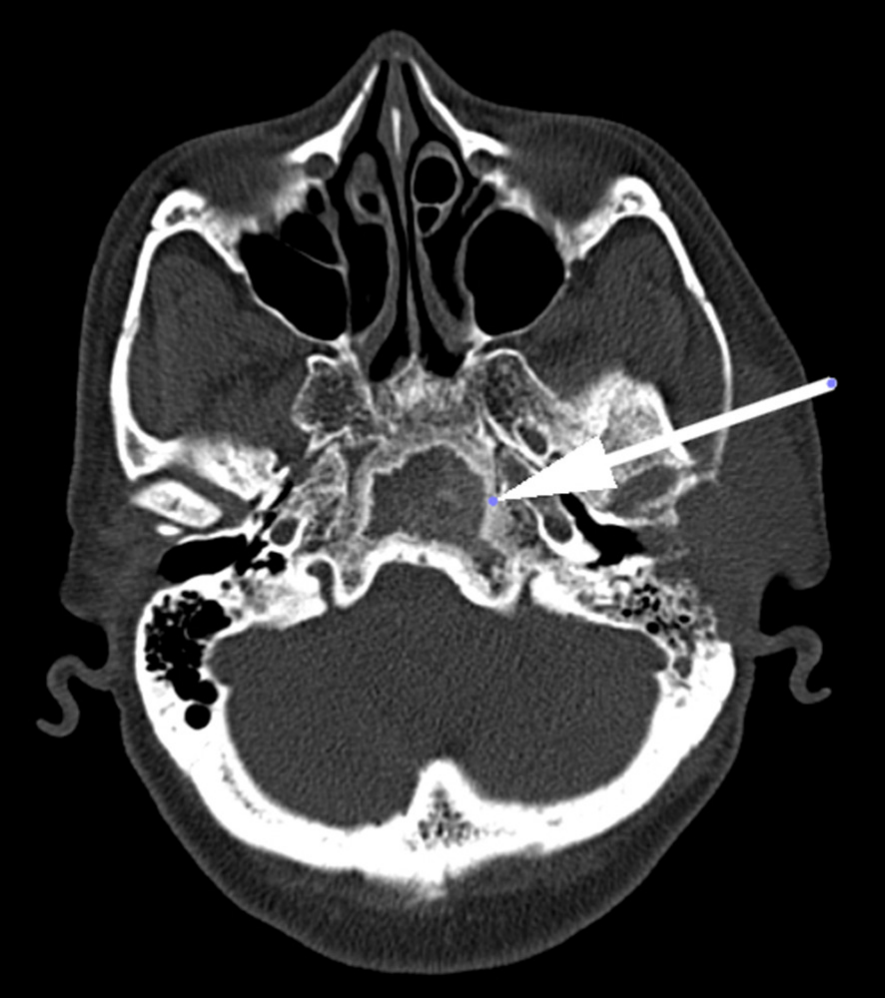
Bone window revealed an expansile, osteolytic lesion of the clivus involving dorsum sellae and the posterior clinoid process (arrow).

**Figure 3.**
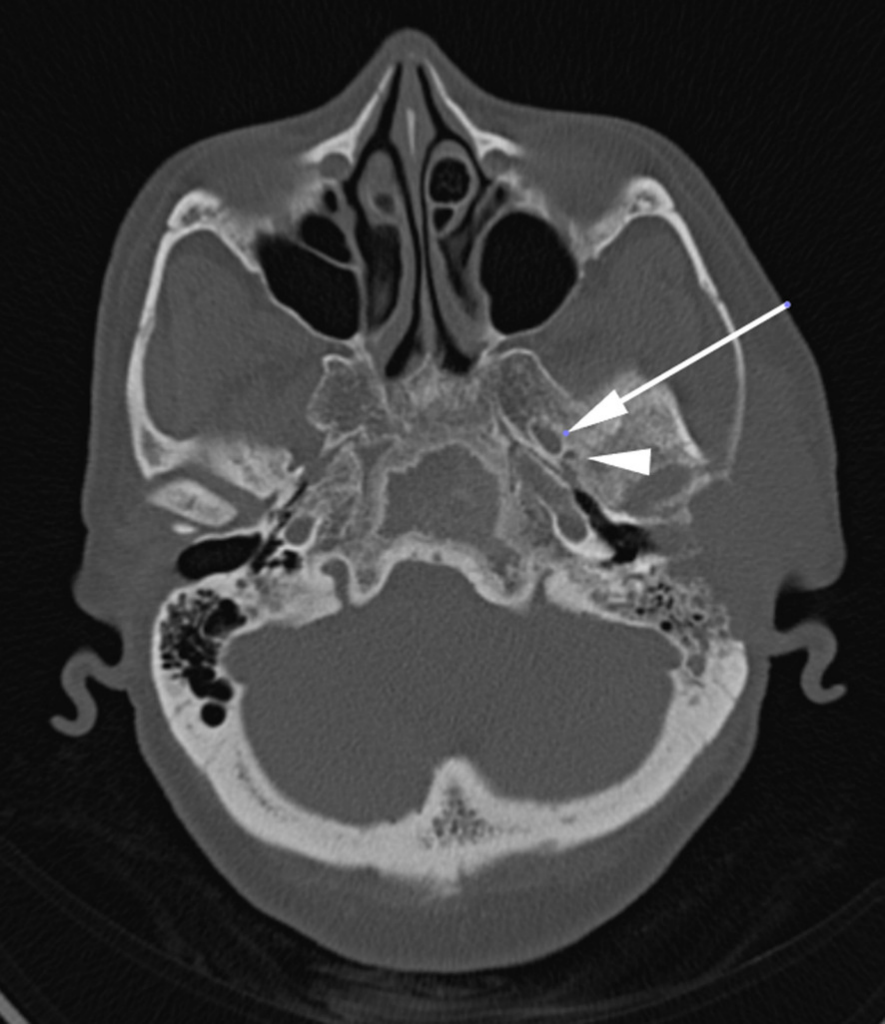
CT axial image bone window shows no bony sclerosis or foraminal widening on the left (arrows).

**Figure 4.**
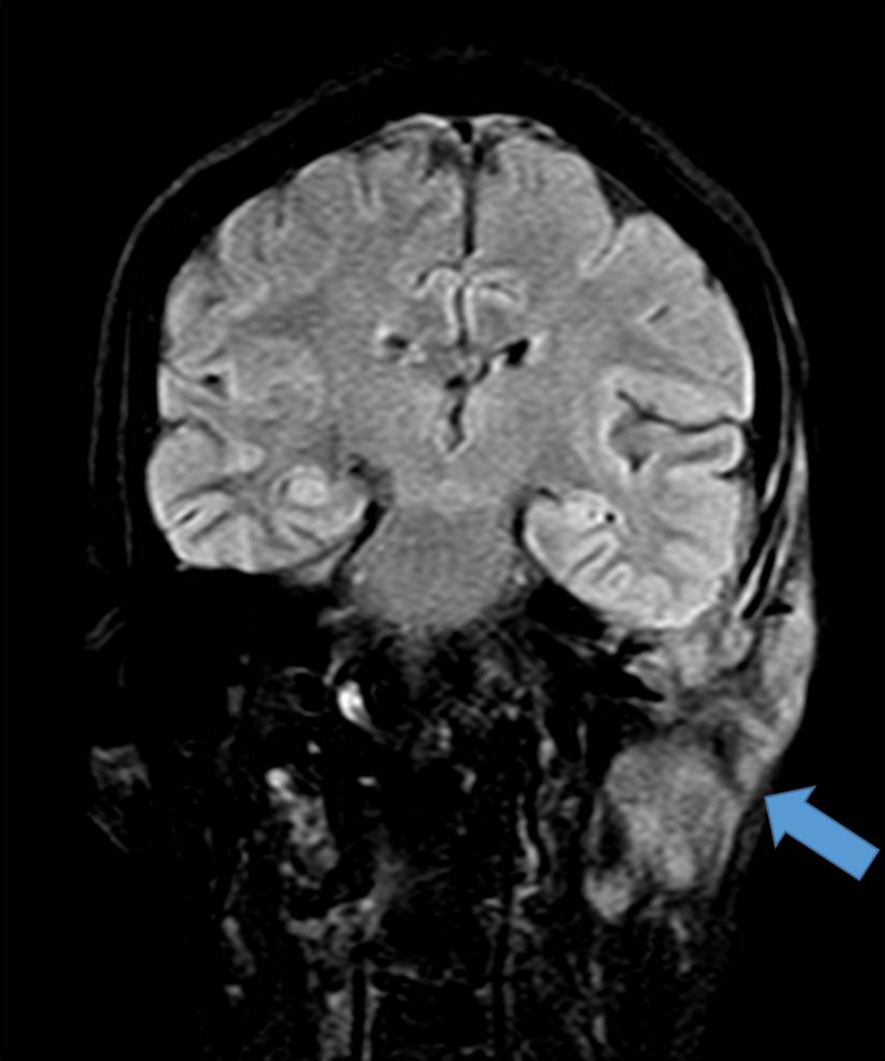
MR shows a hyperintense lesion in the pre- and postauricular region (arrow).

**Figure 5.**
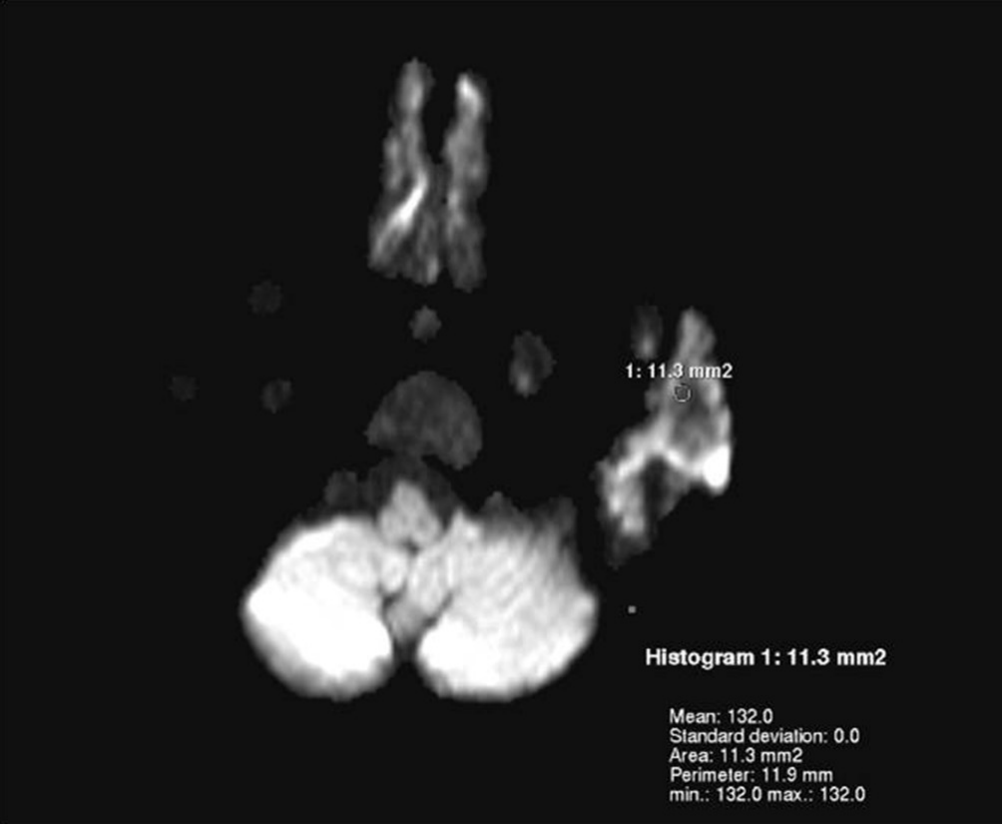
Apparent diffusion coefficient map shows a value of 0.132 × 10^−3^ mm^2 ^s^−1^ favouring a malignant lesion.

**Figure 6.**
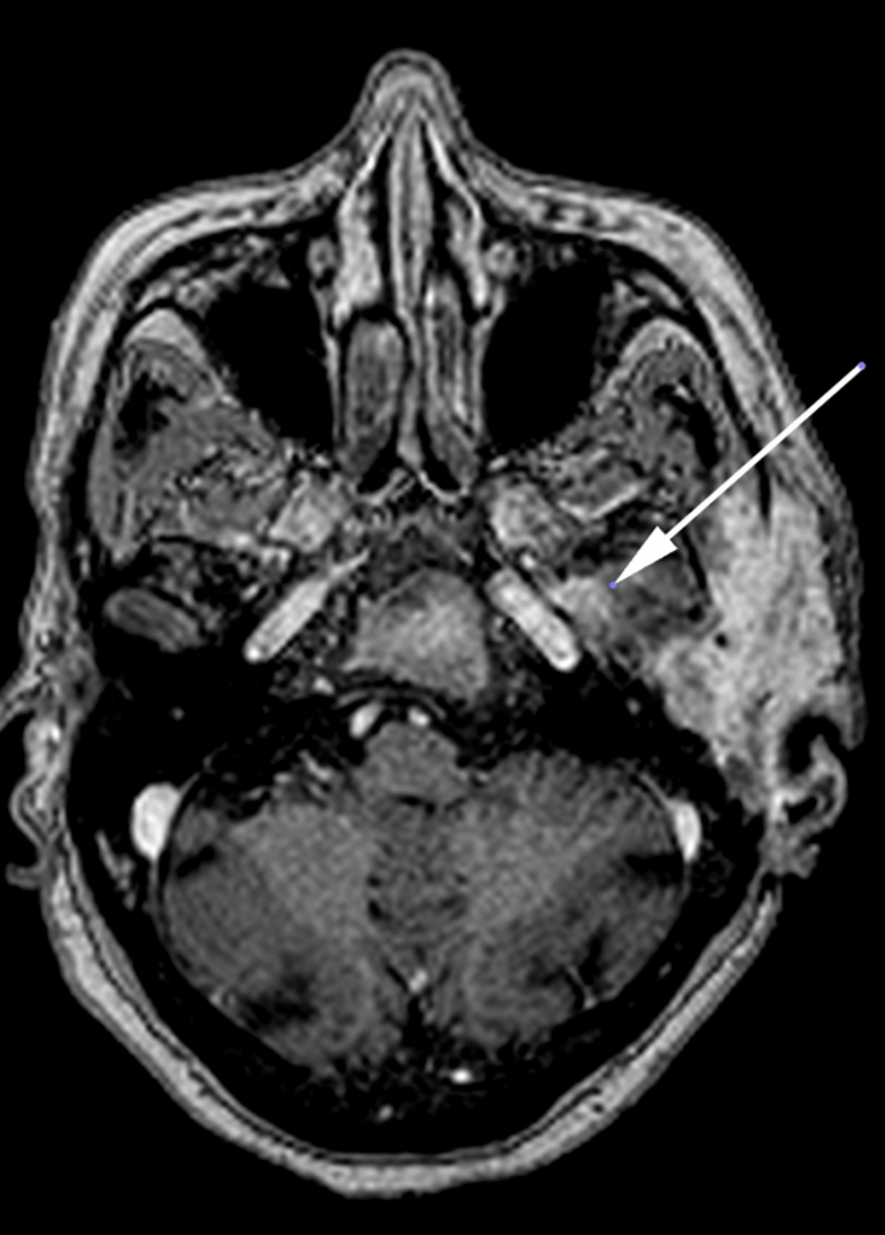
*T*_1_ weighted contrast-enhanced axial MR image with fat saturation shows enhancement of the left foramen ovale and spinosum (arrow), indicating perineural spread *via* the mandibular branch of the left trigeminal nerve.

**Figure 7.**
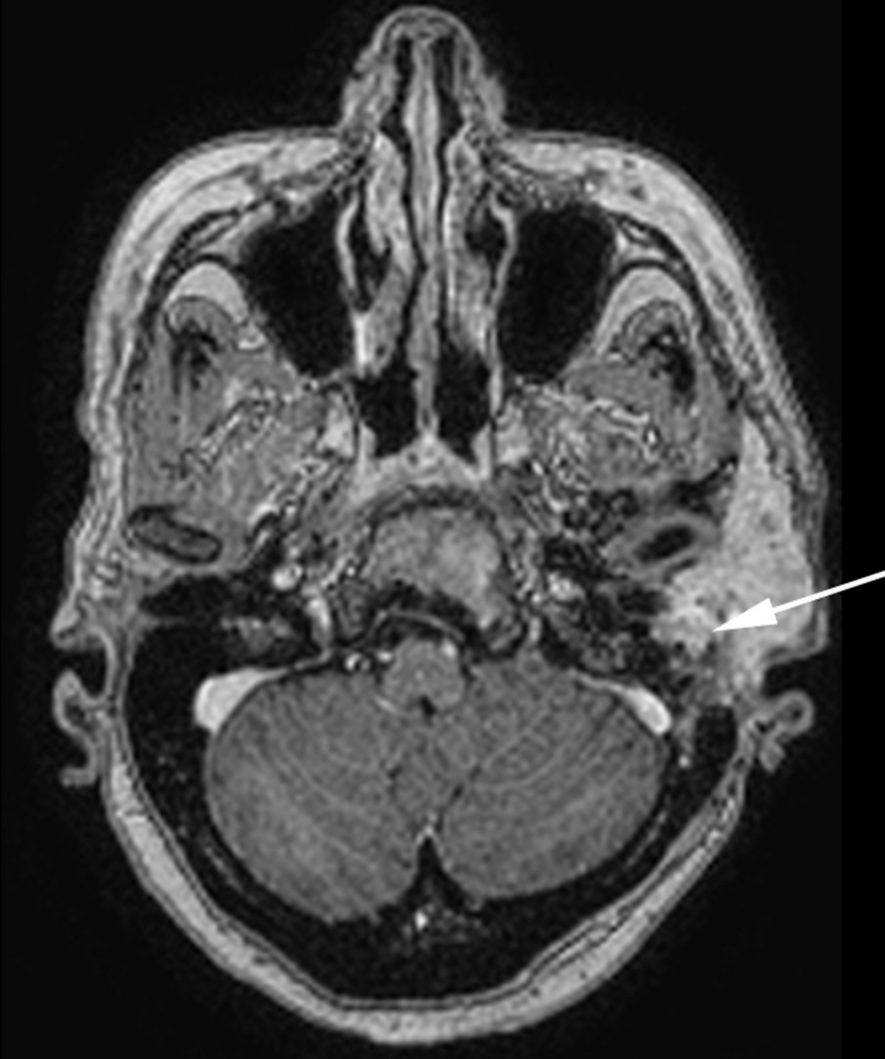
*T*_1_ weighted contrast-enhanced axial MR image with fat saturation shows the lesion involving the region of the left stylomastoid foramen (arrow), indicating perineural spread *via* the facial nerve.

**Figure 8.**
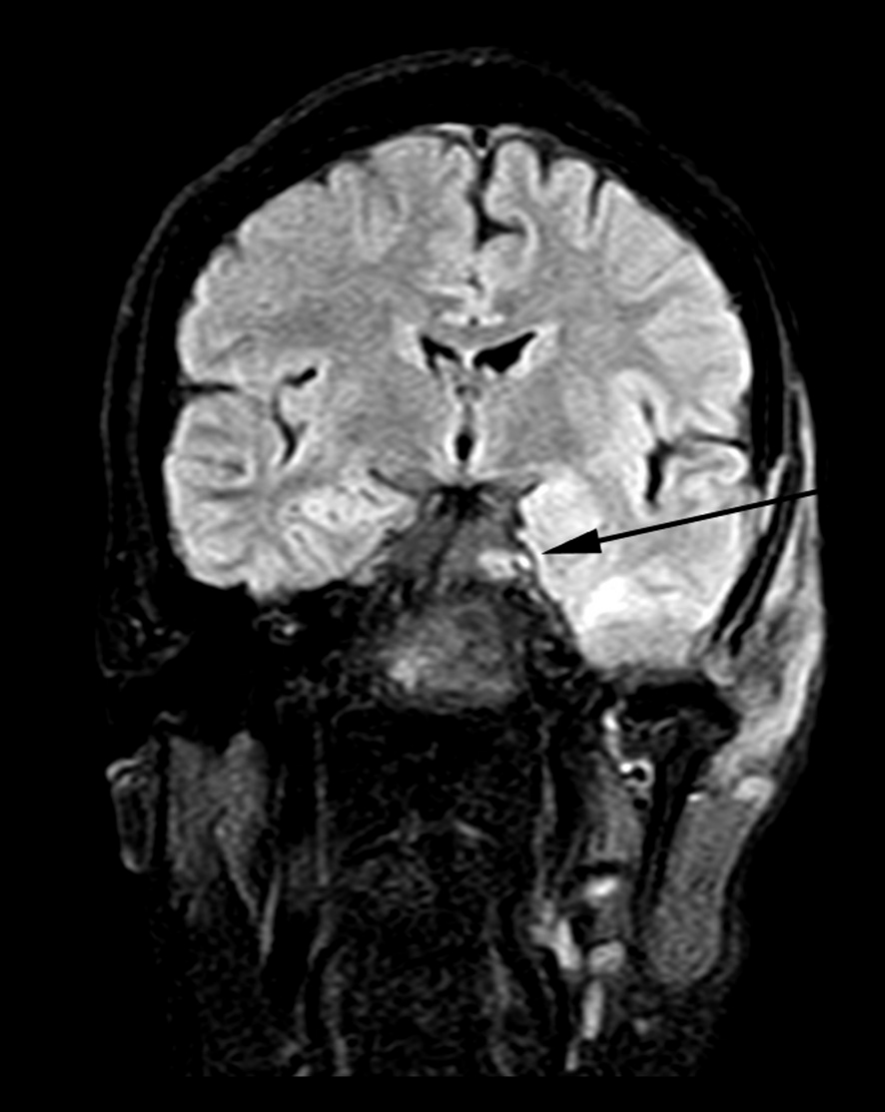
*T*_2_ weighted fluid-attenuated inversion-recovery coronal image shows hyperintensity in the left Meckel's cave (arrow), indicating perineural spread *via* the left trigeminal nerve.

CT contrast study of the neck revealed an ill-defined, homogeneously enhancing lesion in the left preauricular region of the scalp measuring 57 × 21 mm, extending into the left masticator space, causing erosion of the squamous part of the temporal bone with intracranial extension into the temporal lobe region with bilateral level II, III and left VB enlarged lymph nodes (Figure 1). Bone window revealed an expansile, osteolytic lesion of the clivus involving the dorsum sellae and posterior clinoid process (Figure 2). No abnormality was noted in the left foramen ovale and spinosum in the axial section of the bone window (Figure 3). CT thorax revealed parenchymal and subpleural nodules seen in the superior and lateral basal segment of the left lower lobe with an osteolytic lesion involving the D11 vertebra. CT abdomen showed an osteolytic lesion in the left lower ileum extending to the roof of the acetabulum, the right ischium. MRI examination showed a *T*_2_ weighted fluid-attenuated inversion-recovery (FLAIR) hyperintense lesion in the pre- and postauricular region with diffusion restriction and apparent diffusion coefficient (ADC) value of 0.132 × 10^−3 ^mm^2 ^s^−1^ (Figures 4 and 5). Patchy bone marrow oedema was seen in the clivus. *T*_1_ weighted fat-saturated contrast-enhanced MRI shows enhancement in the left foramen ovale, foramen spinosum and stylomastoid foramen (Figures 6 and 7). *T*_2_ weighted FLAIR coronal image shows hyperintensity in the left Meckel’s cave (arrow), indicating perineural spread *via* the left trigeminal nerve (Figure 8).

## Treatment

This patient underwent wide local excision with tumour-free margin and mastoid exploration. Postoperatively, the patient was stable.

## Outcome and follow-up

The patient underwent external beam radiotherapy with a dose of 5000 cGy for a period of 3 months after wide local excision. Subsequently, the patient developed a swelling in the preauricular region with tenderness, and CT and MR examination showed recurrence of the tumour. The patient was then referred to the regional national cancer institute for chemo- and radiotherapy.

## Discussion

ACC is a well-known tumour of the salivary gland and oral cavity that accounts for 10–15% of all head and neck tumours worldwide.^1^ In contrast, primary cutaneous adenoid cystic carcinoma (PCACC) is a rare skin tumour, with <50 cases reported.^2^ PCACC originating in the scalp was first reported by Boggio in 1975.^3^ Other reported locations include the tracheobronchial tree, oesophagus, lacrimal gland, ear canal, breast, uterine cervix, bartholins gland, prostate, mucosal gland of the upper respiratory tract and the skin. PCACC frequently arises in the scalp of middle-aged or elderly patients, with a slight predilection for women.^4^ Histologically, several skin tumours closely mimic PCACC, such as the adenoid cystic variant of basal cell carcinoma, mucinous carcinoma, dermal cylindroma and other primary cutaneous apocrine carcinomas. The lack of epidermal connection, absence of peripheral palisading of neoplastic cells, periodic acid Schiff-positive material and perineural space invasion favour the diagnosis of ACC over other entities. The positivity for carcinoembryonic antigen and S100 protein and negativity for p63 favours the diagnosis of ACC.^5^ ACC of the head and neck is a slow-growing tumour. The average duration of the tumour prior to diagnosis is 9.8 years with a tumour size ranging from 0.5 to 8 cm with an average size of 3.2 cm.^5^ Local recurrences are frequent and metastasis, which occurs in 21–54% of cases, tends to involve the lung, liver, bone and brain.^6–8^ PCACC has a 50% local recurrence rate; however, metastases to regional lymph nodes and distant organs are exceedingly rare. To the best of our knowledge, after extensive literature search, only a few cases of PCACC with distant metastasis have been reported and none with bony metastasis.^9^ Imaging plays a vital role; CT scan determines the spread and analyses the metastatic spread of the disease, as seen in this patient with pulmonary and bony metastases to the vertebra and the iliac bone. The treatment for primary cutaneous ACC consists of either wide local excision or Mohs micrographic surgery.^10–14^ Mohs micrographic surgery has been recognized as the treatment of choice with the highest reported cure rate and very low recurrence rate.^15^ This patient had a recurrence after wide local excision. This could be attributed to the high rate of perineural spread, which accounts for the high recurrence rate after local excision with minimal margins or without micrographic control. ACC has a higher predilection for perineural spread, that is, tumour dissemination along the tissues of nerve sheath. Recognizing perineural spread (Figures 6–8) is of great clinical importance because many of the patients are asymptomatic and very difficult to identify at the time of surgery. It is important to recognize this entity, because it has a detrimental influence on the prognosis and surgical planning. Moreover, patients with perineural spread have poor prognosis and long-term survival. MRI with its superior contrast and spatial resolution has the capability of detecting the different signal intensity of the tumour, fat, cerebrospinal fluid, meninges and brain. This helps in diagnosing perineural spread with 100% sensitivity in MRI as compared with CT, which is evident only at a later stage (Figure 3), with bone erosion, sclerotic margins and widening of normal diameter of the foramina.^16^ We recommend *T*_1_ fat-saturated contrast-enhanced isometric volumetric sequences for identifying perineural spread. *T*_2_ FLAIR sequences are useful in assessing the inflammatory component associated with this entity.

Furthermore, MRI also aids in assessing post-chemotherapy changes by measuring ADC changes following treatment. Sun et al^17^ reported that successful treatment shows early increase in ADC values and ADC changes can be considered as a functional biomarker for monitoring therapeutic response. In addition, diffusion-weighted imaging and ADC value characterize the lesion as benign or malignant. Malayeri et al^18^ demonstrated that ADC values exhibited an inverse relationship with tumour grade. Less aggressive tumours (grade I and *in situ* lesions) showed an average ADC value of >1.5 × 10^−3^ mm^2^ s^−1^ and more aggressive tumours (grade II and III lesions) showed an average ADC value of <1.2 × 10^−3^ mm^2^ s^−1^. Since all tumours follow the same physiology, these ADC values can be used as a reference in this article. The mean ADC value obtained in this lesion was 0.132 × 10^−3 ^mm^2^ s^−1^, favouring the malignant grade. This needs further studying.

Positron emission tomography (PET-CT) acts as a vital imaging modality for assessment and management of head and neck cancer. PET-CT is found to be superior to MR or CT imaging in the assessment of lymph nodes, distant metastasis and synchronous primary malignancy. PET-CT is useful for evaluating the response to treatment and has higher sensitivity and specificity than MRI in detecting recurrent disease.^19–21^ However, our patient did not undergo PET-CT examination.

## Conclusions

PCACC of the scalp is a rare tumour of the head and neck region with common local recurrence but rare distant metastatic spread. Imaging plays a vital role in diagnosis of the primary lesion, recurrence, local and distant metastasis and perineural spread.

## Learning points

1. PCACC of the scalp is a rare skin tumour.
2. It is a locally aggressive tumour with rare lung and bone metastases; hence, imaging of the thorax, vertebral column and pelvis must be performed in patients to rule out metastasis due to the disease. PET-CT is an effective modality in evaluating distant metastasis and is highly recommended.
3. Head and neck tumours are responsible for tumour recurrence and affect the prognosis and long-term survival. MRI is the modality of choice to identify perineural spread, which could result in better planning of surgical approach to achieve tumour-free margin.

## Consent

Written informed consent was obtained from the patient for publication of this case report, including accompanying images.
